# Deep ultraviolet laser direct write for patterning sol-gel InGaZnO semiconducting micro/nanowires and improving field-effect mobility

**DOI:** 10.1038/srep10490

**Published:** 2015-05-27

**Authors:** Hung-Cheng Lin, Fabrice Stehlin, Olivier Soppera, Hsiao-Wen Zan, Chang-Hung Li, Fernand Wieder, Arnaud Ponche, Dominique Berling, Bo-Hung Yeh, Kuan-Hsun Wang

**Affiliations:** 1 Department of Photonics and Institute of Electro-Optics, National Chiao Tung University, 1001 Ta Hsueh Rd., 300 HsinChu, Taiwan; 2 Institut de Science des Matériaux de Mulhouse (IS2M), CNRS - UMR 7361, Université de Haute Alsace, 15 rue Jean Starcky, Mulhouse, France

## Abstract

Deep-UV (DUV) laser was used to directly write indium-gallium-zinc-oxide (IGZO) precursor solution and form micro and nanoscale patterns. The directional DUV laser beam avoids the substrate heating and suppresses the diffraction effect. A IGZO precursor solution was also developed to fulfill the requirements for direct photopatterning and for achieving semi-conducting properties with thermal annealing at moderate temperature. The DUV-induced crosslinking of the starting material allows direct write of semi-conducting channels in thin-film transistors but also it improves the field-effect mobility and surface roughness. Material analysis has been carried out by XPS, FTIR, spectroscopic ellipsometry and AFM and the effect of DUV on the final material structure is discussed. The DUV irradiation step results in photolysis and a partial condensation of the inorganic network that freezes the sol-gel layer in a homogeneous distribution, lowering possibilities of thermally induced reorganization at the atomic scale. Laser irradiation allows high-resolution photopatterning and high-enough field-effect mobility, which enables the easy fabrication of oxide nanowires for applications in solar cell, display, flexible electronics, and biomedical sensors.

Amorphous metal-oxide have emerged as potential replacements for organic and silicon materials in thin-film electronics. The high carrier mobility in the amorphous state, and excellent large-area uniformity, has extended their applications to active-matrix electronics, including displays, sensor arrays and X-ray detectors^1^,^2^,^3^,^4^. Moreover, their solution process ability and optical transparency have opened new horizons for low-cost printable and transparent electronics on plastic substrates. Conventional metal-oxide formation by the sol–gel route requires an annealing step at relatively high temperature, which has prevented the incorporation of these materials with the polymer substrates used in flexible electronics. In past few years, many approaches have been proposed to lower down the sol-gel process temperature^5^,^6^,^7^,^8^,^9^. Among these attempts, ultraviolet (UV) photo-annealing is a promising technique due to effective elimination of organic components and the acceleration of metal-oxide-metal (M-O-M) condensation^10^,^11^,^12^,^13^. The photon energy transferred to sol-gel mixture can initiate the formation of M-O-M network.

When oxide semiconductor thin film is used in various kinds of devices such as transistors, solar-cells or sensors, patterning of oxide semiconductor film is required. Conventionally, photoresist process is used to pattern the oxide thin film. However, when removing photoresist, the acid treatment or plasma treatment usually causes damage on the underlying oxide semiconductor film. Also, because the photoresist residue may cause a poor contact between oxide layer and the following deposited metal electrode, a complete photoresist removal is required. Recently, self-patterning oxide thin-film transistor (TFT) was demonstrated by using deep-ultraviolet (DUV) lamp (main emission peak at 253.7 nm) to irradiate directly on sol-gel oxide precursors through a mask^14^,^15^,^16^. The best resolution of the DUV-photo-patterned indium-gallium-zinc-oxide (IGZO) is 3 μm. The DUV direct-patterning relies on the DUV-induced M-O-M network formation. The condensation reaction in thin film, however, is also easily initiated by thermal heating, even at low temperature (50 °C). The success in photopatterning on sol-gel oxide material hence relies on the sufficient photo-energy transferring without heating up the substrate.

In this work, we firstly demonstrate the ability of DUV laser (wavelength of 193 nm) to write on sol-gel IGZO thin film at room temperature (25 °C). Compared with DUV lamp patterning, the directional DUV laser irradiation avoids the substrate heating and suppresses the diffraction effect. Hence, the line pattern with a 800-nm linewidth and a sharp edge is obtained by mask lithography. Moreover, two-beam DUV laser interference lithography (LIL) is firstly used to direct write IGZO nanowires with width of 300 nm. To evaluate the electrical property of the DUV-laser-write IGZO material, thin-film transistors with DUV laser patterned IGZO channel are fabricated. High-enough electron mobility ranging from 1.1 cm^2^/Vs to 13 cm^2^/Vs can be obtained when the thermal post-annealing temperature varies from 300 °C to 600 °C, which shows that the DUV laser irradiation does not deteriorate the electrical properties. On the contrary, a beneficial effect of the DUV laser was recorded. Atomic force microscope (AFM) images and X-ray photoelectron spectroscopy (XPS) analysis are also performed to investigate the condensation reaction under DUV and thermal annealing. Compared with control device, DUV irradiation is proposed to deliver homogeneous IGZO film with improved mobility.

## Results and Discussion

### DUV-laser-write a-IGZO Patterns

**Figure 1:**
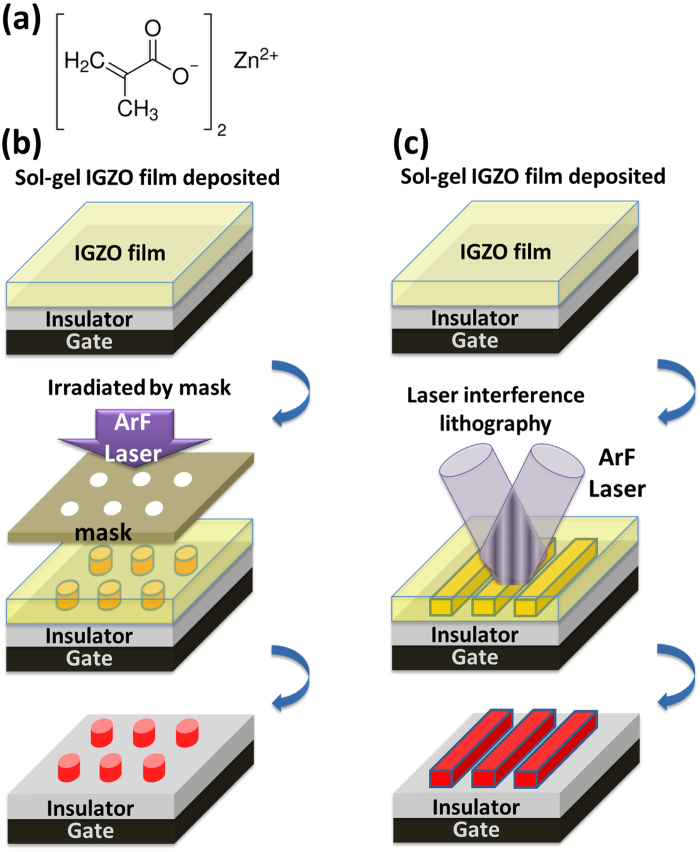
Direct write of IGZO micro and nanopatterns. (**a**) Molecular structure of the zinc methacrylate precursor. Schematic description of patterning methods used including (**b**) patterning by irradiating DUV laser beam on sample through a binary amplitude mask and (**c**) patterning by using interference lithography with DUV laser and phase masks. The material acts as a negative tone photoresist and patterns are obtained after development.

We firstly demonstrate the direct patterning ability of DUV laser irradiation on sol-gel IGZO mixture. The detail of the preparation of IGZO sol-gel mixture is described in *Methods*. A new zinc methacrylate precursor was used in this work. The molecular structure of the zinc methacrylate precursor is given in Fig. 1(a). Zinc methacrylate precursor was chosen because metal methacrylate complexes have been proved to exhibit high sensitivity in the DUV range allowing light-induced crosslinking. Thus they were used as photosensitive building blocks to generate nanopatterning by laser direct write^17^,^18^. The emission wavelength of the DUV laser is 193 nm. The laser density is typically 300 mW/cm^2^ for a beam size of 3 × 6 mm^2^. Two patterning methods are used. (1) Patterning by irradiating DUV laser beam on sample through a binary amplitude mask (names as **DUV-laser-write** hereafter) is as shown in Fig. 1(b). The mask is fused silica substrate with periodic chromium line patterns. The line pattern has two kinds of line widths, 5 μm and 800 nm, while the duty ratio is fixed at 50%. The patterning is carried out in contact lithography configuration to limit the diffraction artefacts. This configuration is possible without damaging the functional film surface nor the mask surface because the film is dry after spin-coating deposition. (2) Patterning by using interference patterns from DUV laser. In this case, a combinaison of two fused silica phase masks is used to generate sinusoidal light patterns as shown in Fig. 1(c). This method is a direct-write interference lithography (IL) process, hence is named as **DUV-IL-write** in this paper. By controlling the interference laser profile, periodic IGZO line pattern can be realized with controlled line width and period. In this work, we fix the period at 600 nm.

**Figure 2:**
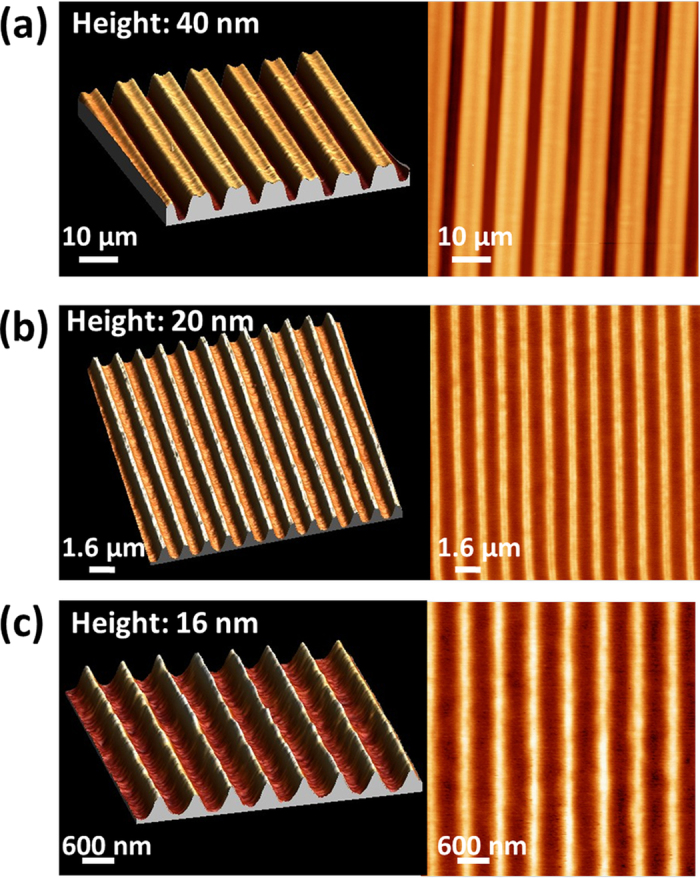
Typical example of IGZO patterns. AFM images of patterns obtained by binary amplitude mask lithography (**a**,**b**) and interference lithography (**c**) Material composition was In:Ga:Zn = 4:1:2. Widths of structures are respectively (**a**) 5 μm, (**b**) 800 nm and (**c**) 300 nm.

The IGZO patterns produced by the two methods are then investigated. The molar ratio of indium, gallium, and zinc is fixed at 4:1:2. The Atomic Force Microscope (AFM) images of the IGZO line pattern with different periods are shown in Fig. 2. Samples in Fig. 2(a,b) are fabricated by using DUV-laser-write while the sample in Fig. 2(c) is fabricated by using DUV-IL-write. We successfully obtain a-IGZO lines with line width ranging from 5 μm to 800 nm by simply irradiating DUV laser through a binary fused silica mask. Moreover, by generating interference patterns with phase masks, we also successfully realize IGZO nanowires with linewidth of 300 nm. The tilted sidewall of IGZO nanowire is due to the sinusoidal light intensity distribution in interference lithography. The writing, however, is effective down to the substrate. One of the major interests of the interference lithography technique is to allow a rapid nanopatterning over large area (few seconds of irradiation for nanostructures over cm^2^). This process opens thus a route to produce nanowire oxide transistor and nanowire oxide sensors.

**Figure 3:**
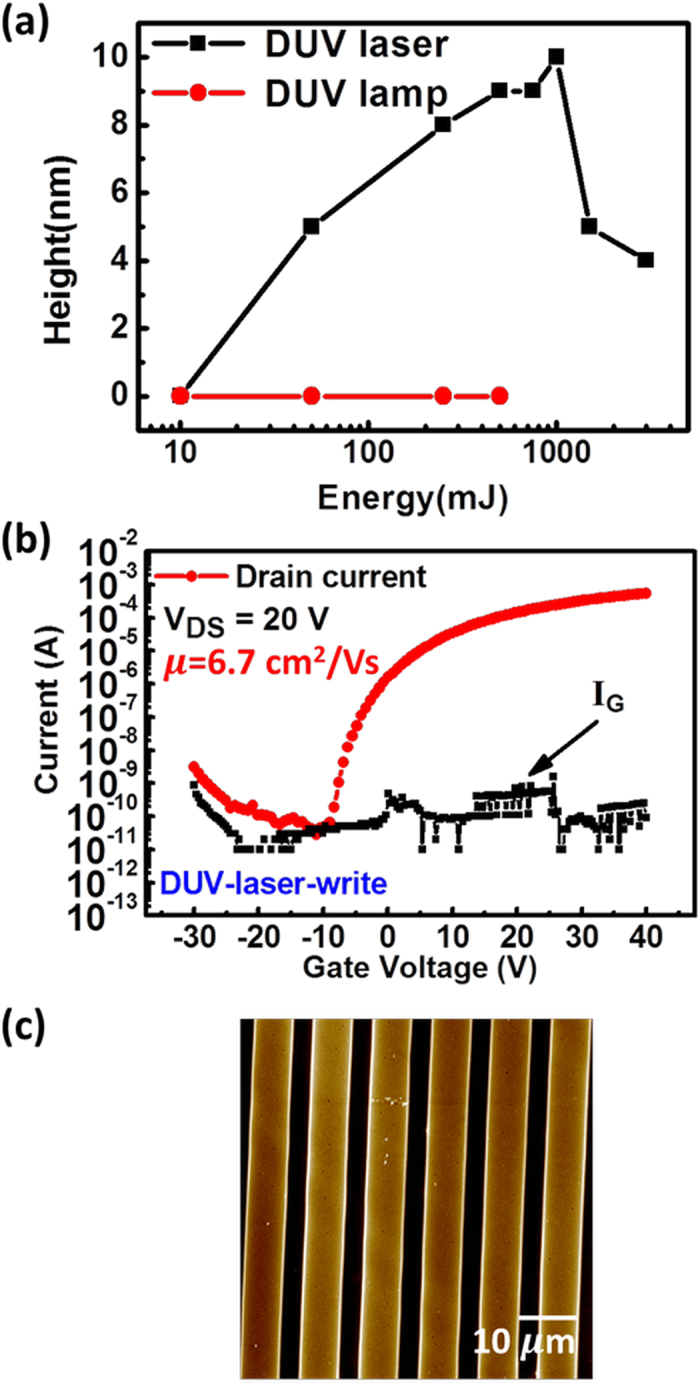
Laser patterning and electrical properties of material. (**a**) The height of IGZO thin film with laser or DUV-lamp irradiation prepared with irradiation through binary mask with line width of 800 nm. (**b**) The transfer characteristic of IGZO TFT with DUV-laser-write periodic multiple channel lines. The thermal annealing temperature is 600 °C. Channel width and length are 1000 μm and 300 μm, respectively. (**c**) AFM image of multiple line channels produced by laser irradiation through binary mask. The period is 10 μm.

The obtained resolution is far better than reported in previous works. The determinant parameters are the use of a DUV laser wavelength (193 nm) and a choice of a suitable precursor accordingly to the emission wavelength. Laser light has the advantage to be directional, which limits the diffraction effects, and opens the possibility to generate regular light nanopatterns by interference lithography. Moreover, it is usually reported that DUV lamp irradiation provokes a heating of the sample up to about 120 °C^10^. Such heating propagates to the parts of the sample protected from light by the mask, inducing unwanted crosslinking. The effect is a dramatic loss of the resolution that can be avoided by using laser irradiation if associated to a suitable precursor. This point is demonstrated by comparing the photoresponse of the IGZO thin film with laser and DUV-lamp irradiation, when a mask with 800 nm width lines is used. The results are plotted in Fig. 3(a). Tuning DUV–lamp irradiation energy from 10 mJ to 500 mJ, no structure is achievable and hence the structure height is 0 nm. Under laser irradiation, the evolution of the structures height is classical for a negative tone resist: at low light dose, no structures are recorded because the crosslinking in bright areas is not sufficient to keep the material on the substrate during development. The maximum height is reached for 1000 mJ. For higher dose, a loss of contrast is recorded due to the crosslinking in the dark area.

The importance of fine patterning, particularly the submicron-meter patterning, can be discussed for two kinds of applications. (1) For display and for electronics applications, the patterning of the oxide active region is required to clearly define the channel region without surrounding current effect. For this purpose, patterning with micrometer resolution is usually enough. A sub-micrometer resolution, however, benefits the future development of oxide transistor with submicron-meter channel length and multiple fin-like channels (i.e. Fin field-effect-transistor (Fin-FET))^19^. (2) For bio/chemical sensor applications, the formation of nanowire transistor is necessary to enlarge the surface to volume ratio and hence to improve the sensitivity. The ZnO nanowire FETs are widely developed for such sensor works^20^,^21^. However, current process still faces challenges for mass production. In this study, the IGZO multi-channel with a linewidth of 300 nm can be directly patterned by interference DUV laser. It is expected that the reported method facilitates the development of IGZO nanowire bio/chemical sensors.

The proposed laser direct write can be used for a wide range of composition of the IGZO precursor. The atomic ratio between In, Ga and Zn in a-IGZO is indeed known to significantly influence the electrical properties of IGZO^22^,^23^. One interest of the solution route for sample preparation is that the composition can be easily modified in a wide range by just adjusting the molar ratio of introduced starting precursors. With this aim, materials with molar ratio of In, Ga and Zn of 6.8:1:2.2, 4:1:2 and 2:1:2 were investigated. In all cases, microphotopatterning can be achieved (Figure S1). The most interesting electrical properties were obtained with a molar ratio of 4:1:2, and thus, the results presented in the following sections are related to this composition.

### Semiconducting properties

After we successfully demonstrate the DUV direct-write IGZO patterns, the electrical property of the direct-write pattern is evaluated by producing DUV-laser-write IGZO TFT with bottom-gate top-contact structure. Firstly, IGZO TFTs with periodic line channels are fabricated. After DUV irradiation, a thermal post-annealing of 600 °C is used to remove the remaining organic moieties (solvant and ligands) and to form the M-O-M network. AFM images (not shown) confirm that the post-annealing only reduces the pattern thickness without changing the line width. The resulted thickness is about 20 nm. Fig. 3(b) shows the transfer characteristic of the DUV direct-write IGZO TFT with periodic multiple line channels. The period of the multiple line channels is 10 μm as shown in Fig. 3(c). The field-effect mobility as high as 6.7 cm^2^/Vs and the threshold voltage of -4.9 V are obtained, verifying the feasibility of the DUV direct write process.

**Figure 4:**
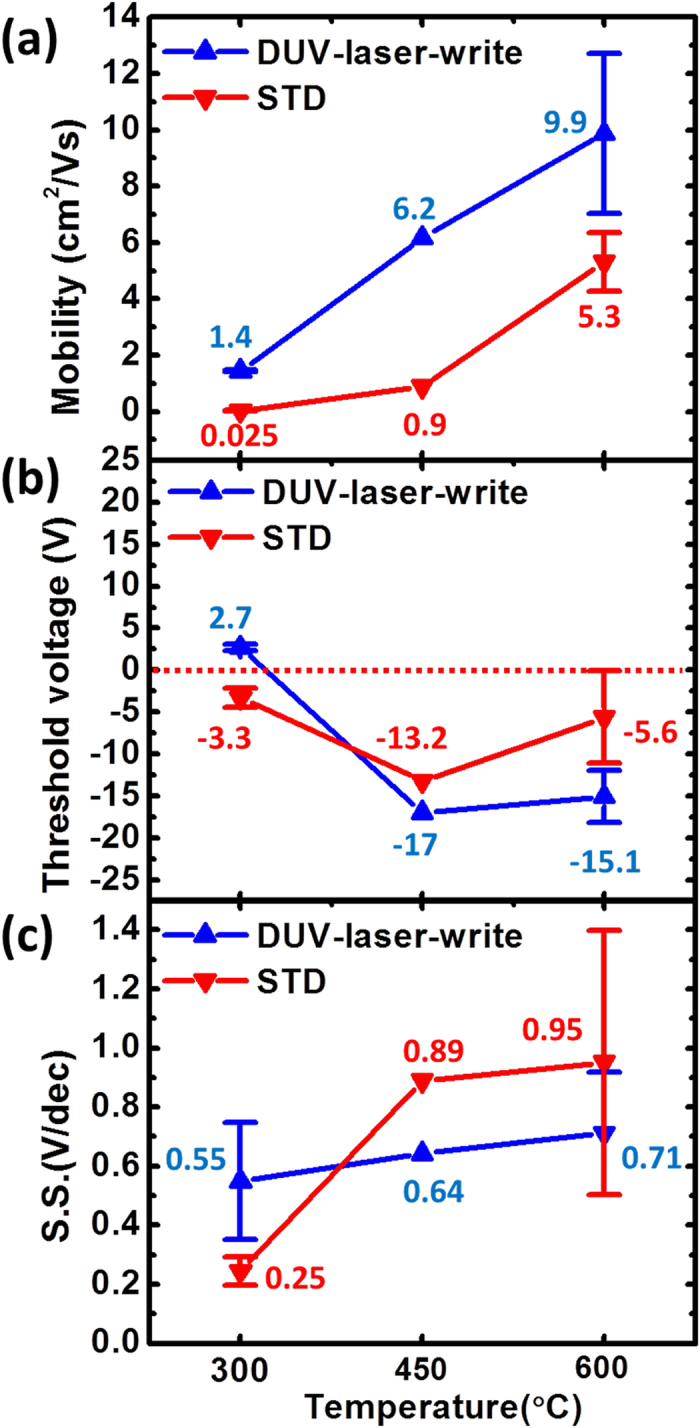
Effect of DUV annealing on electrical properties. (**a**) Mobility, (**b**) threshold voltage, and (**c**) subthreshold swing of DVU-laser-write and of STD IGZO TFTs are plotted as a function of post thermal annealing temperature. Average value and standard deviation are extracted from three independent devices with identical condition.

Then, the influence of DUV laser irradiation on IGZO TFT performance is investigated. IGZO TFTs with and without DUV laser write process are fabricated. Those devices without DUV laser write are named as STD IGZO TFT. The details of the fabrication process are described in *Methods*. The DUV laser irradiation condition is fixed at 2 J and the post-annealing temperature is changed from 600 °C, 450 °C, to 300 °C. The transfer characteristics of DUV-laser-write IGZO TFTs with three different post-annealing temperatures are compared in Figure S2(a). Those of STD IGZO TFTs are shown in Figure S2(b). A good switching property is obtained for DUV-laser-write IGZO TFT. For both DUV-laser-write and STD IGZO TFTs, increasing the post-annealing temperature can effectively increase the output current. The extracted saturation-region field-effect mobility, threshold voltage, and subthreshold swing of DUV-laser-write and STD IGZO TFTs are compared in Fig. 4(a–c), respectively. The parameter extraction method is described in *Method*. The average value and the standard deviation are obtained by measuring three independent devices with identical process condition.

Figure 4(a) shows that the field-effect mobility is significantly increased with post-annealing temperature. The mobility obtained for STD devices with this material is lower than the mobility reported in previous works on sol-gel IGZO TFTs^10^,^16^. The new zinc precursor, zinc methacrylate, may not decompose as well as other precursors when the annealing temperature is as low as 300 °C. However, with DUV irradiation, the mobility can be significantly improved. In Fig. 4(a), the field-effect mobilities of DUV-laser-write IGZO TFTs (blue symbols) are significantly higher than those of STD IGZO TFTs (red symbols). The DUV-enhancement effect is more pronounced when the annealing temperature is reduced. Specifically, when annealing temperature changes from 600 °C, 450 °C, to 300 °C, the mobility enhancement ratios (i.e. the mobility of DUV-laser-write IGZO TFT divided by the mobility of STD IGZO TFT) are about 2, 7, and 56, respectively. It is noted that when decreasing post-annealing temperature from 600 °C to 300 °C, the threshold voltages of DUV-laser-write IGZO TFT also change from a negative value (i.e. -15.1 V) to be close to zero (i.e. 2.7 V). The detail mechanism to explain the shift of threshold voltage in sol-gel oxide TFT is still not well understood. Low post-annealing temperature may avoid the interaction between the densifying oxide film and the ambient gas species including oxygen and water vapor^15^,^24^. In Fig. 4(c), with increasing annealing temperature, we also observe a significant increase in subthrehsold swing of STD device. This phenomenon will be discussed later with material analysis. With 300 °C post-annealing, DUV-laser-write a-IGZO TFT exhibits a field-effect mobility of 1.4 cm^2^/Vs, a threshold voltage of 2.7 V, and a subthreshold swing of 0.55 V/dec. The low enough subthreshold swing indicates that the DUV-laser-write IGZO TFT exhibits low defect density at IGZO/insulator interface as well as in IGZO bulk. The obtained mobility (1.4 cm^2^/Vs) is already higher than the mobility in commercial amorphous silicon (a-Si:H) TFT (typically 0.5 cm^2^/Vs). With the close-to-zero threshold voltage and the low enough subthreshold swing, the proposed DUV-laser-write a-IGZO TFT is promising for developing low-power solution-processed pixel circuitry.

The mobility obtained in this work is also compared with the mobilities reported by other works on sol-gel oxide TFTs in Table S3 in supporting information^5^,^6^,^9^,^10^,^11^,^16^,^25^,^26^,^27^,^28^,^29^,^30^,^31^,^32^,^33^,^34^,^35^,^36^,^37^,^38^,^39^,^40^. There are several reports on oxide TFT using various approaches to improve the mobility as high as 140 cm^2^/Vs^37^. These approaches include the plasma treatment^26^,^27^, the incorporation of conductive nanotube/nanowire^30^,^36^,^37^, and the introduction of new precursor and new compound^16^,^34^,^35^,^38^, etc. Without using these treatments, the mobility reported on sol-gel IGZO TFT is typically 0.0007-0.8 cm^2^/Vs with 300 °C annealing temperature^11^,^25^,^26^ , 0.02-1.56 cm^2^/Vs with 450 °C annealing temperature^29^,^30^,^31^,^32^,^33^, and 6.415 cm^2^/Vs with 600 °C annealing temperature^25^. In our work, STD devices exhibit mobility of 0.025 cm^2^/Vs, 0.9 cm^2^/Vs, and 5.3 cm^2^/Vs with annealing temperature of 300 °C, 450 °C, and 600 °C, respectively. DUV-laser-write devices exhibit mobility of 1.4 cm^2^/Vs, 6.2 cm^2^/Vs, and 9.9 cm^2^/Vs with annealing temperature of 300 °C, 450 °C, and 600 °C, respectively. The mobility obtained in our work is comparable to the reported typical value, verifying that the DUV laser patterning is a feasible approach to deliver good enough electrical performance.

### Material analysis

To investigate the influence of DUV irradiation on sol-gel IGZO, several material analysis methods such as XPS, AFM, FTIR, and ellipsometry analyses are performed on sol-gel IGZO films with and without DUV laser irradiation.

**Figure 5:**
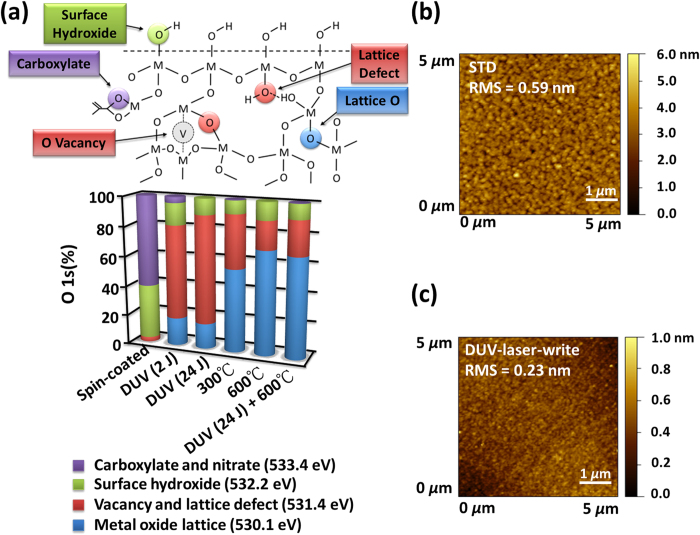
Impact of DUV and thermal treatmant on material structure. (**a**) Schematic representation of different types of oxygen atoms present in the material and their relative proportion determined by XPS after spin-coating, DUV irradiation with 2 J and 24 J, thermal annealing at 300 °C and 600 °C and DUV irradiation followed by thermal annealing (600 °C). Raw XPS spectra and results from deconvolution are given in Figure S4. The AFM image of surface of (**b**) samples prepared by thermal annealing and (**c**) that of samples prepared by DUV annealing.

The reactions within the sol-gel matrix under photochemical and thermal annealing are investigated by XPS. To investigate the condensation reactions activated by photochemical and thermal processes, we first focus on the O 1 s components. The raw spectra and details of the deconvolution are given in Figure S4. Basically, one can find on each samples, 4 main components that are attributed according to data from literature^24^: O of carboxylate and nitrate species (533.4 eV), O of surface hydroxide (532.2 eV), O near an oxygen vacancy or O of volume hydroxide in defects, with interaction (H bonding for example) (531.4 eV), O of metal oxide lattice (530.1 eV). The results issued from the deconvolution are given in Fig. 5(a) and Table S5.

After spin-coating, as expected, oxygen atoms are mostly contained in carboxylate, nitrate and hydroxide groups, coming from metal precursors and partially hydrolyzed precursors. The formation of inorganic oxide lattice can not be detected at this stage, showing that the condensation reaction in the solution is inhibited. This explains the good stability with time of the IGZO precursor solution. The presence of many polar groups is also important to ensure a good adhesion of the film during spin-coating and especially to avoid dewetting effect.

The laser irradiation deeply modifies the material, even for lower dose (2 J) corresponding to conditions for DUV patterning. First, the contribution of oxygen from ligands disappears, which corresponds to the photolysis of the starting complexes. Zn-carboxylates, In-nitrates and Ga-nitrates are decomposed under DUV light. Such photochemical pathway was already described for other transition metal like Zr, Ti or Hf linked to methacrylate acid^17^. N 1 s XPS data given in Figure S6 confirms the loss of the 408.5 eV peak, characteristic of nitrates. Main contribution after laser irradiation is the 531.4 eV peak. Considering the loss of the ligands of the metal precursors, we attributed this contribution mainly to oxygen of hydroxides embedded in the thin film. This oxygen can be bonded by hydrogen bonds, which accounts for the crosslinking of the material used for photolithography. Interestingly, the formation of the metal oxide lattice is not really increased when the dose is increased from 2 to 24 J. However, after DUV laser irradiation, the level of defects (hydroxides) in the material remains too high to confer any interesting electrical properties. This demonstrates that the DUV laser curing is very efficient to provoke the crosslinking of the IGZO precursors needed for direct writing but further condensation necessary to reach the optimal electrical properties is obtained only after a thermal post-annealing step.

Thermal curing of IGZO precursors thin film was also investigated at 300 °C and 600 °C. With thermal annealing, the prevailing contribution corresponds to the oxygen from the metal oxide lattice. The yield of metal oxide lattice is noticeably higher when the temperature is increased from 300 °C to 600 °C. The corresponding loss of defects from further condensation probably accounts for better electrical properties of the sample cured at higher temperature. A significant decrease of the 531.4 eV peak is also observed. Such evolution is the result of decrease of hydroxide embedded in the film and increase of oxygen vacancy. The level of hydroxide groups at the surface of the material remains unaffected by the thermal treatment, since dehydration of metal oxide only occurs at higher temperatures.

Combination of photochemical (24 J) and thermal treatment leads, for oxygen, to the same state as for only thermal treatment. The difference in electrical properties are thus not due to a further conversion into metal oxide, but probably, as discussed previously, because the DUV irradiation leads to a cold mineralization that produces a freezing of the material into a more homogeneous distribution, preventing segregations.

This assumption was supported by AFM characterization. The AFM images of sol-gel IGZO with and without DUV laser irradiation are compared in Fig. 5(b,c), respectively. The post annealing temperature is 600 °C. The surface roughness of IGZO without DUV laser irradiation is 0.59 nm while that of IGZO with DUV laser irradiation is only 0.23 nm. In Fig. 5(b), several large particles are observed, indicating a severe aggregation during the thermal annealing if no DUV treatment is carried out before this step. The severe aggregation may also explain the large subthreshold swing (i.e. 0.95 V/dec in Fig. 4(c)) of STD device with 600 °C post annealing. It is well accepted that the metal ion may diffuse during thermal annealing. The conductive indium aggregation at back interface may deteriorate the gate depletion function and hence cause a poor subthreshold swing. For sol-gel IGZO with 300 °C post annealing without DUV treatment, no aggregation can be observed from the AFM image in Figure S7 in supporting information. As a result, the subthreshold swing of STD device with 300 °C post annealing is as good as 0.25 V/dec as shown in Fig. 4(c).

The DUV-induced modifications were also investigated by spectroscopic ellipsometry and FTIR. The absorption spectra of the starting material given in Figure S8(b) confirm the absorption of the starting material at the excitation wavelength (193 nm). The absorption band covers a wide range of wavelength that allows the use of DUV lamps also for photocrosslinking, as shown below. Interestingly, an important shift of the absorption spectrum during DUV irradiation is observed demonstrating the photolysis of the absorbing species. The photolysis is confirmed by the FTIR spectra (Figure S8(c)). A decrease of the main bands comprised between 1200 and 1650 cm^−1^ is observed. Such bands are usually attributed to organic ligands bounded to metal^41^. Their progressive disappearance upon DUV irradiation confirms the photolysis of the starting precursors, as observed in XPS^17^. The decrease of the wide asymmetric band with maximum at 3400 cm^−1^ corresponds to the loss of C-H of organic ligands. The new band at 1050 cm^−1^ can be assigned to the metal oxide network. Finally, these data are consistent also with the evolution of the thickness and refractive index of the film that reveals a densification of the material under DUV irradiation. This behavior is due to the condensation reaction and release of photoproducts. However, the densification induced by DUV irradiation is not complete and the thermal annealed samples undergo a further densification due to the condensation of the hydroxide pending groups as shown by XPS.

## Conclusion

In summary, we successfully demonstrated the direct write technology of a sol-gel IGZO film by utilizing DUV laser lithography. A formulation of DUV-sensitive precursors for preparing IGZO film was developed and a direct-write structure down to 300 nm was produced by keeping the 25 °C substrate temperature during DUV laser irradiation. The DUV-direct-write technology was also used to pattern the active layer in sol-gel oxide TFT. With 300 °C post-annealing, the DUV direct-write IGZO TFT exhibits field-effect mobility significantly higher than the control device without DUV irradiation. We concluded from the material analysis that DUV irradiation leads to a fast freezing of the material into a more homogeneous distribution without segregations, hence delivering an enhanced mobility than those of the controls. The reduced surface roughness with DUV irradiation also supports the proposed mechanism. The DUV laser interference direct-write enables the easy fabrication of oxide nanowires for applications in solar cell, display, flexible electronics, and biomedical sensors.

## Methods

### Sol-gel solution preparation and sol-gel film coating

The sol-gel IGZO solution was prepared by dissolving indium nitrate hydrate (In(NO_3_)_3_•H_2_O, Aldrich), gallium nitrate hydrate (Ga(NO_3_)_3_•H_2_O, Aldrich), and zinc methacrylate (Zn(CH_2_CH_3_COO)_2_, Aldrich) precursors in 2-methoxyethanol (CH_3_OCH_2_CH_2_OH, Aldrich). The molar ratio of In, Ga, and Zn was 4:1:2 while the total molar concentration of metals is 0.25 M. All solutions were stirred for 1 day at room temperature before spin-coating on the substrate. Heavily doped Si wafer was prepared as the bottom gate and the 100-nm-thick thermal silicon nitride (SiNx) was used as the gate dielectric. Samples were firstly treated with UV/Ozone for 10 minutes. IGZO films were spin-coated on the substrate with 4000 rpm for 40 s.

### Standard IGZO TFT fabrication process

After IGZO film was spin-coated on the substrate, 130 °C pre-annealing for 1 minute was used to remove the solvent. Then, samples were placed into an ambient furnace with temperature of 300 °C, 450 °C, or 600 °C for 1 hour. After thermal annealing, we put a dry photo resist film onto the sample and use UV light to irradiate the dry photo resist through a shadow mask. Then, after developing the dry photo resist by using KOH (5%) and etching the unwanted IGZO region by using HCl (99%), IGZO active region was formed. The dry photo resist was removed by acetone. Finally, a 100-nm-thick aluminum was thermal evaporated through shadow mask to form the source and drain electrodes. The channel width is defined by the width of IGZO active region as 1000 μm and the channel length is defined by the distance between the source and drain electrodes as 300 μm.

### DUV-laser-write IGZO TFT fabrication process

After IGZO film was spin-coated on the substrate, DUV irradiation was used to pattern the IGZO film. For DUV-laser-write devices, DUV laser (ArF laser with emission wavelength of 193 nm) was used to irradiate the IGZO film through a fused silica mask to form the active layer as periodic multiple lines or through a shadow mask to form a circular spot (with diameter of 3 mm). The non-irradiated part was removed by developing the samples in 2-MOE for 10 s. Then, samples were placed into an ambient furnace with temperature of 300 °C, 450 °C, or 600 °C for 1 hour. Finally, a 100-nm-thick aluminum was thermal evaporated through shadow mask to form the source and drain electrodes. The channel length is defined by the distance between the source and drain electrodes as 300 μm or 50 μm.

### Electrical measurement and material analysis

Agilent E5270B semiconductor parameter analyzer was used to measure the electric characteristics of IGZO-TFT in room temperature. By plotting the square root of the drain current (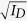) as a function of gate voltage (V_G_) under saturation condition, the threshold voltage and mobility were extracted from the x-axis intercept and the slope of the plotted curve.

XPS analysis was performed on a Gammadata Scienta (Uppsala, Sweden) SES 200-2 X-ray photoelectron spectrometer under ultra-high vacuum (P < 10^−9^ mbar). The monochromatized AlKa source (1486.6 eV) was operated at a power of 420 W (30 mA and 14 kV) and the spectra were acquired at a take-off angle (TOA) of 90° (angle between the sample surface and photoemission direction). During acquisition, the pass energy was set to 500 eV for wide scans and to 100 eV for high-resolution spectra. CASAXPS software (Casa Software Ltd, Teignmouth, UK, www.casaxps.com) was used for all peak fitting procedures and area of each components were modified according to classical Scofield sensitivity factors (Zr3d: 7.04, C1s: 1.00 and O1s: 2.93). All components on high-resolution spectra were referenced according to the CH_x_ component at 285.0 eV.

## Additional Information

**How to cite this article**: Lin, H.-C. *et al.* Deep ultraviolet laser direct write for patterning sol-gel InGaZnO semiconducting micro/nanowires and improving field-effect mobility. *Sci. Rep.* **5**, 10490; doi: 10.1038/srep10490 (2015).

## Supplementary Material

Supplementary Information
