# Supplementary material for: Deep ultraviolet laser direct write for patterning sol-gel InGaZnO semiconducting micro/nanowires and improving field-effect mobility
Source: Sci Rep. 2015 May 27;5:10490. doi: 10.1038/srep10490 (PMC4444848; doi:10.1038/srep10490)
Supplement: Supplementary Information [file srep10490-s1.pdf]

Deep ultraviolet laser direct write for patterning sol-gel InGaZnO semiconducting micro/nanowires and improving field-effect mobility

Hung-Cheng Lin, Stehlin Fabrice, Olivier Soppera*, Hsiao-Wen Zan*, Chang-Hung Li,

Fernand Wieder, Arnaud Ponche, Dominique Berling, Bo-Hung Yeh, Kuan-Hsun Wang

Supplementary Information

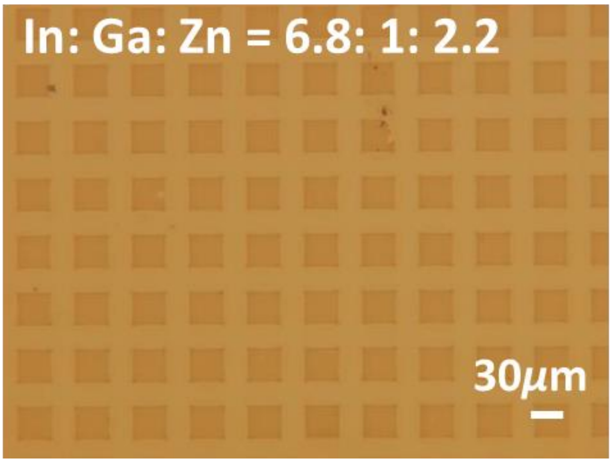

Figure S1 | The optical image of periodic IGZO squares patterned by using the DUV laser write. The square are is 30×30 μm^2 and the molar ratio between In, Ga, and Zn is 6.8:1:2.2.

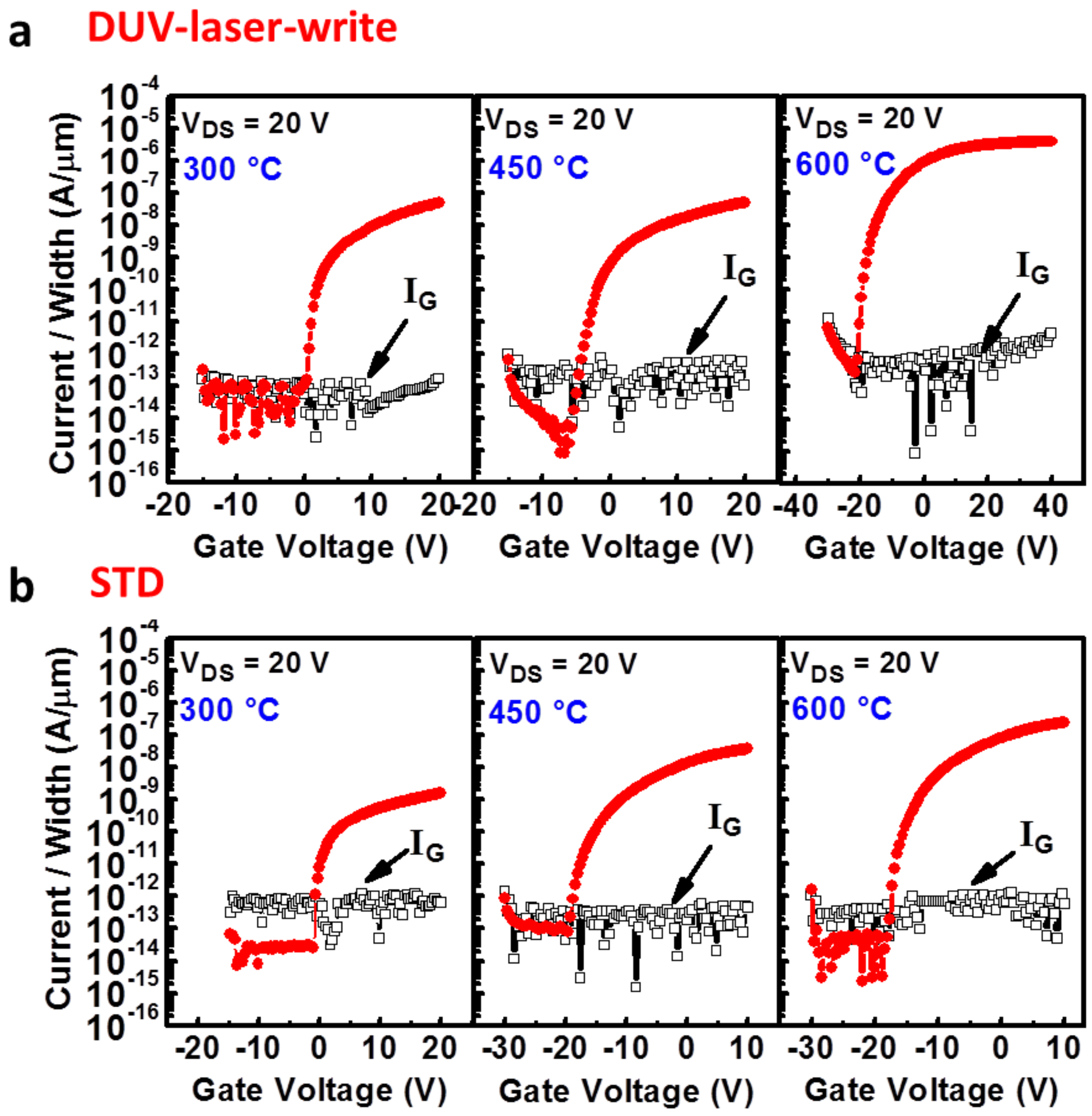

Figure S2 │ The transfer characteristics of (a) DUV-laser-write IGZO TFTs and of (b) STD IGZO TFTs. Devices with post annealing temperature as 300℃, 450℃, and 600℃ are compared. Channel length is fixed as 300 μm.

Ref.	Semi-conductor	Annealing temperature(℃)	μ(cm^2/Vs) (without treatment)	μ(cm^2/Vs) (enhanced)	Treatment
Our work	IGZO	300<br>450<br>600	0.025<br>0.9<br>5.3	1.4<br>6.2<br>9.9	DUV direct patterning
5	IZO<br>IGZO	230-275<br>275	<0.1<br>N/A	7-12<br>4.05-8.78	hydrolysed annealing
10	IGZO<br>IZO<br>In_2O_3	350	6.01<br>3.72<br>10.31	8.76<br>4.43<br>11.29	DUV annealing
11	IGZO	300<br>350<br>400	0.0007<br>0.0081<br>0.6	2.45<br>N/A<br>N/A	assisted with UV-ozone annealing
16	IGZO	350	2.83	15.51	direct light pattern integration with AcAc doping
25	IGZO	300<br>400<br>500<br>600	0.003<br>0.065<br>2.1<br>6.415	N/A	N/A
26	IGZO	300<br>350<br>400	0.80<br>2.80<br>4.30	2.70<br>7.20<br>11.20	oxygen plasma treatment
27	IGZO	400	0.072	1.07	oxygen plasma treatment
28	IGZO	220<br>250	0.05<br>N/A	1.81<br>3.13	high-pressure annealing
29	IGZO	450	1.21	2.17	multistacked active layer
30	IGZO	450	0.02	0.17	single-walled carbon nanotube (SWNT) doping
31	IGZO	450	0.42	N/A	N/A
32	IGZO	450	0.32	2.09	Dual-Active-Layered Zinc-Tin-Oxide (ZTO)/IGZO
33	IGZO	450	1.56	22.16	confining the ITZO layer into IGZO
34	IGZO	350<br>400	N/A<br>N/A	3<br>10-20	different precursors
35	IGZO<br>In_2O_3	250<br>350	N/A<br>1.40	2.24<br>1.15	UV Photopatterning with BzAc or AcAc doping
6	In_2O_3	300<br>500	3.8<br>55.26	16.76<br>N/A	annealing in O_2/O_3 environment
9	InBa(Sr)ZnO	200-225<br>450	N/A<br>5.91-11.80	4.4<br>18-25	dipping in water before annealing in the air
36	ZnMgO	350	N/A	130	SWNT doping
37	IZO	350	1.9	140	SWNT doping
38	IZO	320	2.7	32.6	In_2O_3 nanocrystal doping
39	IZO	250<br>275<br>300	7<br>4.5<br>12	N/A<br>11<br>N/A	with additional vacuum annealing
40	ZTO	500	2.8	5.6	soft annealing

Table S3 | Comparison of recent works on sol-gel oxide TFTs

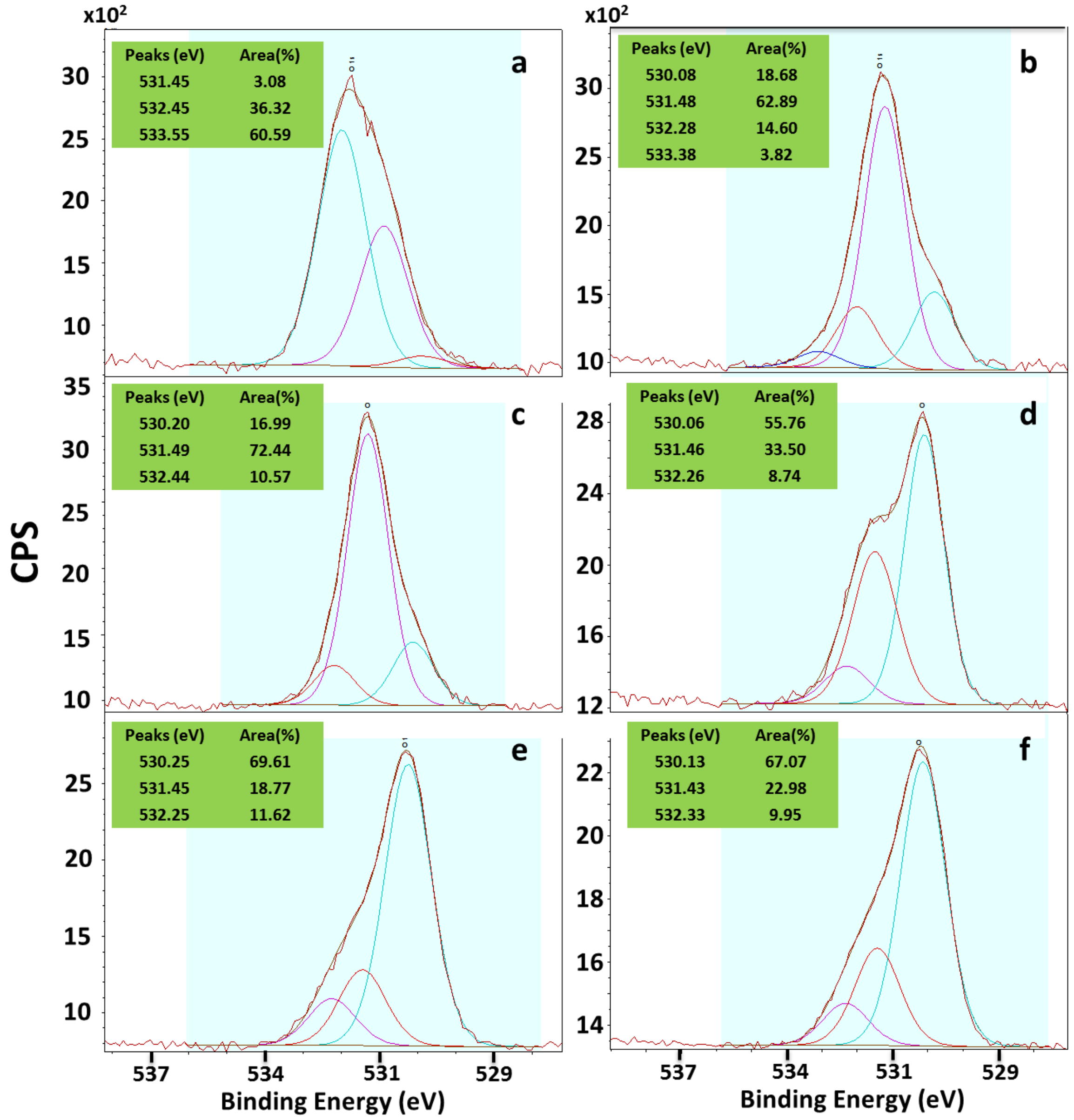

Figure S4 │ O1s high resolution XPS spectra of IGZO precursor material (In:Ga:Zn=4:1:2) (a) after spin-coating, (b) after DUV irradiation (2 J), (c) after DUV irradiation (24 J), (d) after thermal annealing (300℃, 1 h), (e) after thermal annealing (600℃, 1 h), (f) after DUV irradiation (24 J).

Sample condition	Metal oxide lattice (530.1 eV)	Vacancy and lattice defect (531.4 eV)	Surface hydroxide (532.2 eV)	Carboxylate and nitrate (533.4 eV)
Spin-coated	0	3.08	36.32	60.59
DUV(2 J)	18.68	62.89	14.6	3.82
DIV(24 J)	16.99	72.44	10.57	0
300℃	55.76	35.5	8.74	0
600℃	69.61	18.77	11.62	0
DIV(24 J)+ 600℃	67.07	22.98	9.95	0

Table S5 │ Relative proportion of different oxygen atoms present in the material with several preparation methods, from O 1s XPS spectra analysis.

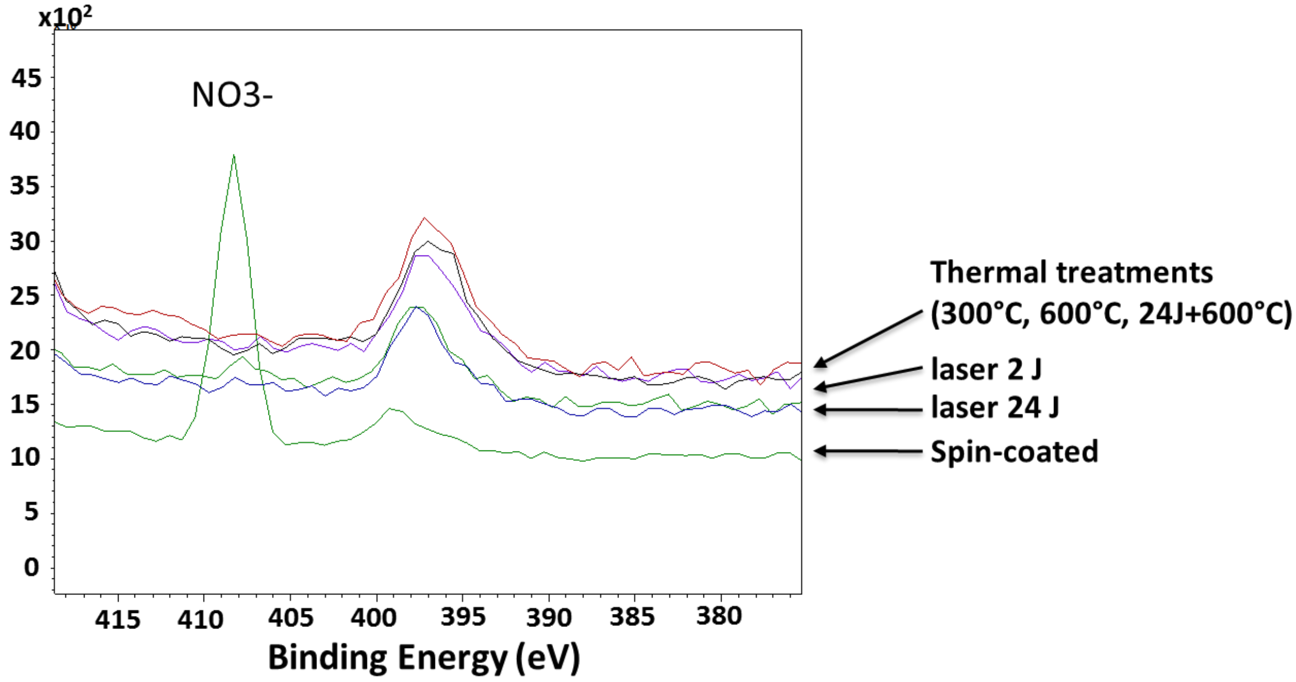

Figure S6 │ N1s XPS spectra of samples prepared by spin-coating and DUV and/or thermal annealing.

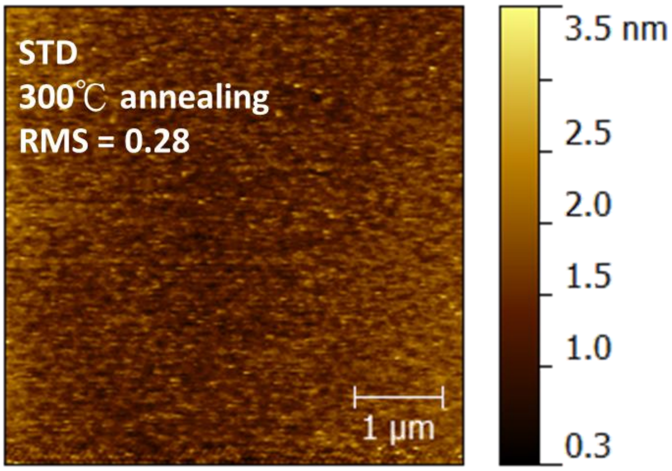

Figure S7 | AFM image of device prepared by thermal annealing of 300℃

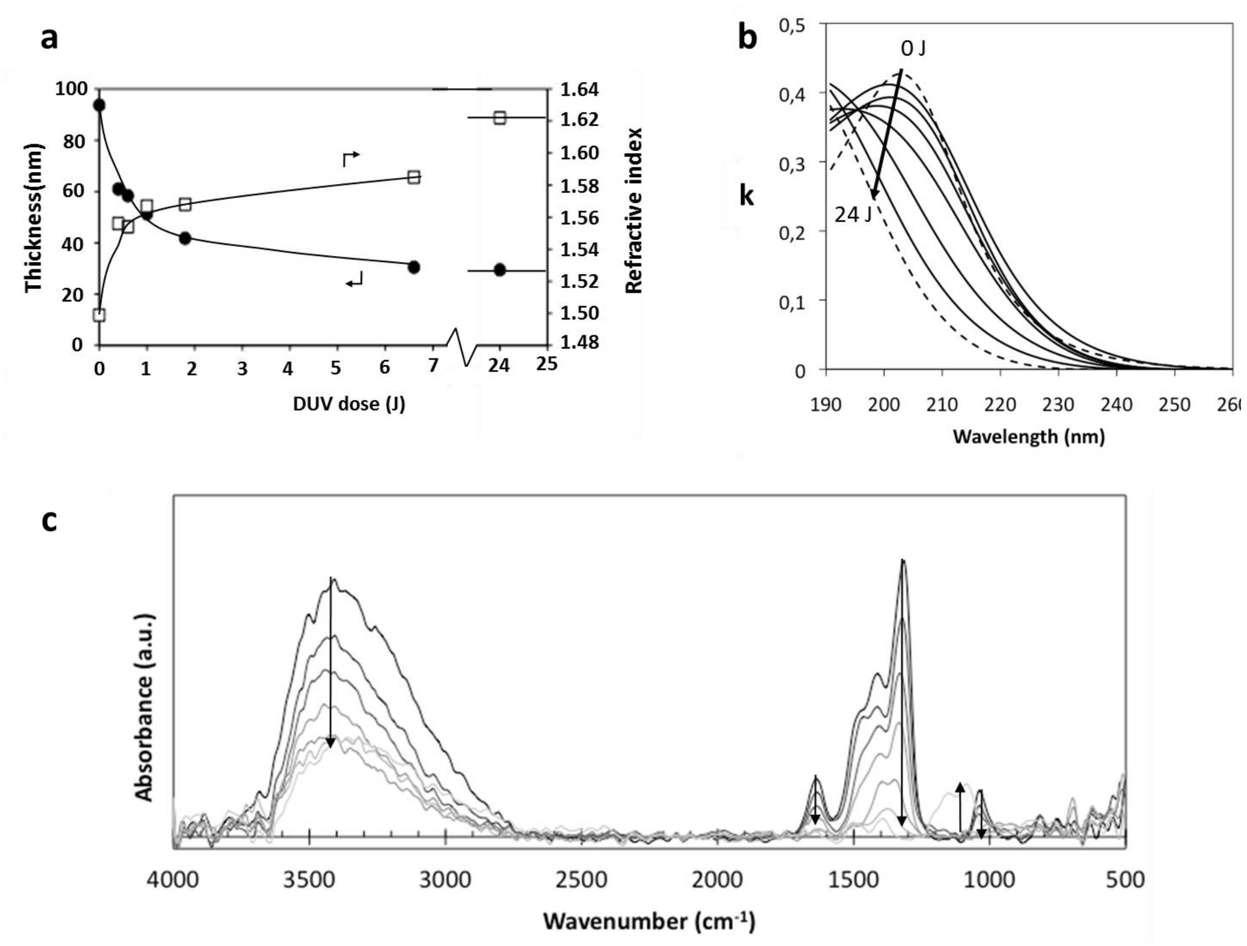

Figure S8 │ (a) Thickness and refractive index of the IGZO thin film during DUV irradiation recorded by spectroscopic ellipsometry. (b) Evolution of extinction coefficient with DUV irradiation. (c) Evolution of FTIR spectrum with DUV irradiation. Samples were irradiated with dose ranging from 0 to 24 J.
